# Routine use of punch biopsy to diagnose small fiber neuropathy in fibromyalgia patients

**DOI:** 10.1007/s10067-014-2850-5

**Published:** 2014-12-24

**Authors:** Todd D. Levine, David S. Saperstein

**Affiliations:** Phoenix Neurological Associates, Ltd, 5090 N 40th Street, Suite #250, Phoenix, AZ 85018 USA

**Keywords:** Epidermal nerve fiber density, Fibromyalgia, Skin punch biopsy, Small fiber neuropathy

## Abstract

Fibromyalgia is a clinical syndrome that currently does not have any specific pathological finding to aid in diagnosis. Therefore, fibromyalgia is most likely a heterogeneous group of diseases with similar symptoms. Identifying and understanding the pathological basis of fibromyalgia will allow physicians to better categorize patients, increasing prospective treatment options, and improving potential therapeutic endeavors. Recent work has demonstrated that approximately 50 % of patients diagnosed with fibromyalgia have damage to their small unmyelinated nerve fibers. A skin punch biopsy is a sensitive and specific diagnostic test for this damage as a reduction in nerve fiber density allows for the diagnosis of small fiber neuropathy. Small fiber neuropathy is a disease with symptoms similar to fibromyalgia, but it often has a definable etiology. Identifying small fiber neuropathy and its underlying cause in fibromyalgia patients provides them with a succinct diagnosis, increases treatment options, and facilitates more specific studies for future therapeutics.

## Introduction

Fibromyalgia (FM) is the second most commonly diagnosed rheumatic disorder and is prevalent in 2 to 8 % of the population [1–3]. FM has been described as a clinical syndrome without any specific pathological findings to confirm a diagnosis. The diagnosis of FM is particularly challenging as it commonly presents as a constellation of ill-defined symptoms producing a heterogeneous group of diseases with similar complaints [4]. To add to the challenge, the most recent diagnostic criteria for FM mandates excluding all other disorders that could account for the pain [1].

For many patients, FM is a lifelong disorder, which many sufferers describe as being in a state of chronic pain. Although the centralized nature of the pain implies that it originates in, or is amplified by, the central nervous system, it does not rule out peripheral nociceptor input as a contributing factor to the pain. In fact, FM patients may experience more pain than typically expected from the contributing nociceptive input [3]. The subjective and non-specific symptoms of FM make the diagnosis and treatment a challenge for the clinician, which undoubtedly leads to frustration for patients. In addition, patients who experience neuropathic symptoms often do not receive the most accurate diagnosis or appropriate treatment. This lack of validation of their symptoms leads to further stress [3].

While FM is a syndrome with unidentifiable causes and pathophysiology, small fiber neuropathy (SFN) is a well-defined disorder with an identifiable pathogenesis and distinct underlying causes [4–7]. Symptoms of SFN usually present distally, manifesting as foot or leg pain. As SFN advances, the symptoms can spread proximally and involve the torso as well [4]. Typical symptoms of SFN include paresthesia, allodynia, hyperesthesia, and numbness. Patients usually describe these sensations as painful, using terms such as burning or shooting. SFN patients often exhibit decreased pinprick sensation, hyperalgesia, or reduced thermal sensation in affected areas. However, sensory examination can be entirely normal in patients with SFN [5]. Additionally, skin changes of the affected area such as shiny skin or decreased moisture of the skin surface that leads to cracking may also be observed [4, 6].

Nerve fibers vary in size and function, with large myelinated A-alpha and A-beta fibers transmitting signals for proprioception and touch, while small myelinated A-delta fibers and unmyelinated C fibers transmit signals for pain and temperature. SFN is caused by dysfunction and degeneration of the small unmyelinated C fibers and the thinly myelinated A-delta fibers [4]. The most frequent underlying cause of SFN is diabetes mellitus [8, 9], with other causes including impaired glucose tolerance, vitamin deficiency (especially B12), hepatitis C virus, human immunodeficiency virus, vasculitis, celiac disease, Sjorgen’s syndrome and other autoimmune conditions, hematological malignancies, infections, toxins (alcohol, medications), and genetic mutations [5–8]. These various conditions cause deterioration of the small nerves under the skin, leaving them damaged or dead, which then results in transmission of abnormal signals, and ultimately produces the burning or shooting pain associated with SFN [5, 8, 10, 11].

Despite clear pathophysiology and known etiologies, diagnosis of SFN in patients with pure SFN (no damage to the large nerve fibers) is challenging because motor coordination, reflexes, light touch, proprioception, and vibratory sensation often appear normal during examination [6]. Although physical examination and medical history of the patient have been the gold standard used to diagnose SFN, ancillary testing may provide added guidance. Some of the available tools for testing have included the neuropathic pain inventory, quantitative sensory testing (QST), quantitative sudomotor axon reflex testing (QSART), electromyography, and nerve conduction studies. Additionally, another diagnostic technique that has recently become widely and commercially available is the skin punch biopsy, which is used to measure epidermal nerve fiber density (ENFD) [6].

## FM/SFN diagnosis

Previously, the American College of Rheumatology (ACR) diagnostic criteria (1990) for FM was based on the presence of widespread pain and tender points [12]. However, this definition of FM was controversial as changes in the amount of pain or number of tender points could vary over time, causing patients previously diagnosed with FM to no longer qualify for a FM classification [1, 13]. In 2010, the ACR updated the FM criteria based on the Widespread Pain Index (WPI) and Symptoms Severity Scale [SS; [1]]. However, neither of these diagnostic criteria included any objective testing, forcing most clinicians to rely on subjective clinical complaints to diagnose their FM patients. Due to the subjective diagnostic criteria, unknown causes, and non-specific symptoms, many clinicians consider FM to be a collection of ill-defined illnesses. SFN is one such condition that, in the absence of an established objective diagnostic test, had been placed under the generic diagnosis of FM. Patients with SFN have complaints of tingling, burning, and pain, which often is not recognized as a distinct condition and continues to be misinterpreted as FM. In many cases, the patients’ symptoms are symmetrical, persistent, and length-dependent, resembling mixed-fiber neuropathy. However, some patients experience symptoms that are non-length-dependent such as inconsistent numbness that is multifocal [11]. With the most prominent symptom of SFN being only the “burning” pain, many patients describe no unique feature to distinguish SFN from other disorders. Diagnosis of SFN, therefore, occurs largely by means of exclusion [7] similar to FM as evidenced by one of the main criteria for diagnosis of FM being ‘lack of a disorder that could explain the pain’ [1]. Although some clinicians utilize multiple diagnostic tests for the diagnosis of neuropathic pain, many tests have limited sensitivity and specificity for SFN. The physiological characteristics of small nerve fibers make diagnosis with electrophysiological tests particularly difficult [5]. Therefore, it is not surprising that diagnostic testing using electromyography and nerve conduction studies were found to be predominantly insensitive for SFN [4].

To fill in the void of objective diagnostics for SFN, the skin bunch biopsy technique was developed at the Karolinska Institute and later standardized at the University of Minnesota and at Johns Hopkins University. These punch biopsies began to be included in the diagnostic workup of patients with suspected SFN after the identification of antibodies against protein gene product 9.5 (PGP 9.5). PGP 9.5 is a neuronal form of ubiquitin carboxyl-terminal hydrolase transported with the slow component of the axonal transport. The availability of this antibody enabled visualization of the extensive innervation of the epidermis [5, 14] and the capacity for quantitation of epidermal innervation by small fibers [9]. This skin biopsy test was so specialized, however, that it remained solely in research hands for many years. It requires skilled and highly trained pathologists to process the sample and perform the very detailed task of manually counting the nerve fibers. It has, however, recently become commercially available at many academic centers and a few specialized labs in the USA. Each lab has its own established cutoff values for ENFD (lab norms), which allow the practitioners to make a diagnosis of SFN with greater certainty [15]. The convenience and ease of modern day shipping methods have made this test readily available to clinicians all over the country.

Over the past few years, skin punch biopsy testing has emerged as a diagnostic standard for SFN. This diagnostic procedure is recommended in practice guidelines for the diagnosis of SFN by both American and European task forces [10, 15]. The European Federation of Neurological Societies has provided a level A recommendation for the use of skin biopsy to measure the density of small fiber epidermal innervation [15]. Additionally, an American Polyneuropathy Task Force also recommended the use of skin biopsy in the evaluation of polyneuropathies, which include SFN [10]. The joint task force guideline report, published in 2010, updated their guidelines to include skin punch biopsies in the diagnosis of SFN, noting that, based on the vast experience of skin punch biopsies in 10 established laboratories worldwide, the 3-mm skin punch biopsy technique is a safe and minimally invasive procedure [15]. Major side effects have not been reported with the skin punch biopsy. Only 2 side effects were reported, the first being mild infections, often resulting from improper wound management which resolved easily with topical antibiotic therapy. Excessive bleeding from the punch biopsy was the only other side effect reported, which resolved without the need of a suture [15].

One of the key benefits of the skin biopsy test is its effectiveness in assessing SFN when compared to other diagnostic tests, such as electromyography and nerve conduction studies. The test can also be repeated multiple times to monitor disease progression or treatment efficacy [16]. Specificity and sensitivity reported for the skin punch biopsy vary from 88 to 92 % [15]. Another benefit of early skin biopsy testing for SFN in FM patients is that patients and their clinicians can have objective evidence explaining the origin of the symptoms. Understanding the pathological basis for the patient’s neuropathic symptoms will allow clinicians to categorize patients more effectively. In addition, an accurate diagnosis of SFN can prompt an in-depth assessment to uncover any underlying diseases as a possible root cause of the patient’s symptoms. Table 1 provides a list of evaluations that may be performed to determine the underlying etiology of SFN in these patients [17]. Identifying the underlying etiology of SFN can also lead to better treatment plans, testing for new and more effective treatment choices, and promoting research to develop new therapeutics. Incorporating the skin biopsy test as a routine evaluation for all FM patients may be beneficial in diagnosing SFN in approximately 50 % of patients. Because the skin biopsy test can reliably demonstrate the loss of epidermal nerve fibers, it can not only confirm the diagnosis of SFN when clinical and neurophysiologic examinations are not adequately informative but also indicate progression of the disease by observing the reduction of ENFD over time with repeated skin biopsies [15].

**Table 1:** Potential causes of small fiber neuropathy and related testing

Causes of small fiber neuropathy	Evaluation
Diabetes, impaired glucose tolerance	2-h glucose tolerance, HgbA1c
Sjogren’s syndrome	SS-A, SS-B, salivary gland biopsy
Lupus connective tissue disease	ANA, ANCA
Sarcoidosis	ACE level
Vitamin B12 deficiency	B-12, methylmalonic acid, homocysteine
Celiac disease	Gliadin and transglutaminase antibody
HIV	HIV serology
Paraprotein/amyloid	Serum immunofixation, quantitative immunoglobulins
Alcohol abuse	History
Chemotherapy	History

*ACE* angiotensin converting enzyme, *ANA* anti-nuclear antibody, *ANCA* anti-neutrophil cytoplasmic antibody, *HgbA1c* hemoglobin A1c, *HIV* human immunodeficiency virus

## Clinical relevance and utility of skin punch biopsy

In a case-controlled study, 25 FM patients were compared with 55 healthy controls matched for gender and age. This study demonstrated a quantitative reduction in epidermal innervation and regeneration sparing myelinated nerve fibers in FM patients [17]. Participants underwent punch biopsies from 2 regions of the leg. For the lower leg biopsy, the median ENFD was 5 fibers/mm in FM patients, compared to 9.5 fibers/mm in the healthy controls. Similarly, for the biopsy sample from the upper thigh, the median ENFD for FM patients was 8.0 fibers/mm compared to the control group with 11.6 fibers/mm. Utilizing the skin biopsy technology, the investigators were able to objectively demonstrate the loss of nerve fibers in some FM patients [18].

Another case-controlled study published in 2013 examined 25 FM patients and 30 gender- and age-matched controls [4]. In this study, participants were evaluated with the autonomic function test (AFT) and the skin punch biopsy. Punch biopsies of 2 to 3 mm in diameter were collected from the standard distal leg site of the participants. The study results revealed that 50 % of the FM subjects and only 17 % of the control subjects had one or more of the objective test results that revealed SFN (*p* < 0.001). In this study, skin biopsy results alone demonstrated that 41 % of FM subjects had ENFD in the <5th percentile of predicted lab norms, in comparison to 3 % of control subjects (*p* < 0.001). The investigators propose that their findings suggest that some FM patients with neuropathic pain could have undiagnosed SFN [4].

More recently, a prospective study of 20 FM patients and 32 age-matched, healthy controls found that 40 % of patients with neuropathic-like symptoms demonstrated decreased ENFD [19]. Another 2014 study of 41 FM patients and 47 healthy controls demonstrated significantly decreased ENFD in FM patients in the calf (5.8 vs 7.4 fibers/mm; *p* = 0.0002) and thigh (9.3 vs 11.3 fibers/mm; *p* = 0.0007) compared to controls [20].

Finally, a retrospective study of 56 FM patients evaluated at specialized peripheral neuropathy centers found reduced ENFD in 61 % of the subjects [18]. Additionally, laboratory evidence for underlying causes of SFN was found for 71 % of the patients with decreased ENFD [18].

## Feasibility of skin punch biopsy

The skin punch biopsy is an easy procedure that can be performed in a doctor’s office by the physician, nurse practitioner, or physician’s assistant. This procedure causes minimal pain and discomfort to the patient requiring no sutures, only an adhesive bandage. The procedure is found to be easier than an electromyogram nerve conduction study that is routinely performed to diagnose neuropathic conditions. The skin biopsy test is a medically necessary and approved test that is fully recognized by the American Academy of Neurology. It is no longer an experimental procedure and is billable and reimbursable through the majority of commercial carriers and Medicare. The ease, speed, and convenience of modern shipping options also make skin punch biopsy testing a feasible option for clinicians.

The utility of punch biopsy in FM has been further validated by a retrospective chart review, which revealed a change in treatment plans in 36 of 69 (52 %) patients after a punch biopsy was performed [21]. Having an accurate diagnosis will make a drastic difference to those patients who have been told that their symptoms have no pathophysiological explanation. Knowing that their skin biopsy revealed a reduced ENFD gives patients an objective and scientific validation of their symptoms. Therefore, it is imperative that skin biopsy be a fundamental part of the routine diagnostic workup of FM, especially in patients with symptoms suggestive of SFN such as burning, stabbing, tingling, and allodynia, in contrast to the typical deep muscular and aching pain seen in FM [4, 18–20].

## Conclusion

Previous studies have demonstrated that approximately 50 % of patients diagnosed with FM may have SFN contributing to their sensory and autonomic symptoms [4, 17, 18], indicating that the measurement of ENFD is an essential step in evaluating FM patients and excluding other treatable causes of their pain. The skin punch biopsy is a simple, reliable, and definitive diagnostic test that is currently available for the measurement of ENFD for these patients [10, 15]. The biopsy can be performed in any physician’s office and shipped at room temperature to one of the few specialized labs that performs this type of testing. The results give physicians an objective diagnostic tool to fulfill the diagnostic criteria for FM by excluding SFN as a cause of their patients’ pain [1] and may allow for the detection of any underlying etiology and ultimately provide appropriate treatment and a possible cure for some of these SFN patients [22]. The skin punch biopsy procedure is no longer an experimental test, it is endorsed by the major neurology and neuropathy associations nationally and internationally, making it reimbursable by most insurance carriers. It is also easily performed in the clinic with minimal discomfort to the patient. The benefits of accurate diagnosis and the ease of testing make the skin punch biopsy an integral part of diagnostic testing for SFN among FM patients.
